# Mental health issues in childhood and adolescence, psychosocial resources and socioeconomic status – An analysis of the KiGGS Wave 2 data

**DOI:** 10.25646/8865

**Published:** 2021-12-08

**Authors:** Claudia Schmidtke, Raimund Geene, Heike Hölling, Thomas Lampert

**Affiliations:** 1 Robert Koch Institute, Berlin Department of Epidemiology and Health Monitoring; 2 Berlin School of Public Health, Alice Salomon Hochschule, Berlin

**Keywords:** MENTAL HEALTH BURDENS, PSYCHOSOCIAL RESOURCES, KIGGS WAVE 2, SOCIAL SITUATION-BASED HEALTH PROMOTION

## Abstract

Mental health burdens are among the most common health issues in childhood and adolescence. Psychosocial resources can act as protective factors and can help in preventing the development and reduce the symptoms of mental health issues. This article discusses this relationship and the availability of these resources within the three different social status groups among 11- to 17-year-olds. The database is the second wave of the German Health Interview and Examination Survey for Children and Adolescents (KiGGS Wave 2, 2014–2017). Mental health issues were assessed via the Strengths and Difficulties Questionnaires; psychosocial resources via self-reported personal, family and social resources; social status was ascertained through a multidimensional index based on the information provided by parents on education, occupational status and income. The analyses show that 11- to 17-year-olds who have psychosocial resources are less likely to show mental health issues (independent of their social status) and that, compared to high social status, mental health issues are more frequently associated with low social status. Children from (socially) worse-off families have less access to resources. The results consequently highlight the importance of prevention and health promotion measures directed at strengthening resources. Focusing such measures on the needs of disadvantaged population groups should contribute to health equity.

## 1. Introduction

The course of a person’s future health is set very early on in life. From a life-course-epidemiology perspective, mental health issues in childhood and adolescence play an important role for health in later life. The risk of issues manifesting as a disorder, becoming chronic and of various comorbidities developing is great [1, 2, 3]. A pronounced social gradient is observed in the occurrence of mental health issues, with an increased risk for children and adolescents from the low-status groups [4, 5].

Psychosocial resources in terms of personal, family and social resources, are of particular importance, as they act as protective factors and are capable of positively influencing mental health. This protection can help in preventing the development of mental health issues or otherwise ensure that children and adolescents with mental health issues nevertheless develop into mentally healthy adults [6]. However, children and adolescents from socially disadvantaged backgrounds are demonstrably less likely to count on these resources than those from socially better-off families.

Also with regard to health equity, the ties between mental health issues, psychosocial resources and social status are key to strengthening health promotion and prevention. Important references here are the target anchored in Germany’s Prevention Act [7, 8] of ‘reducing socially rooted and gender-related inequalities in health opportunities’, the health goal ‘Growing up healthy: life skills, exercise, nutrition’ [8, 9, 10], which is also mentioned in the Prevention Act, as well as the Cooperation Network on Equal Health Opportunities [11].

The German Health Interview and Examination Survey for Children and Adolescents (KiGGS) provides data on the physical and mental health of children and adolescents, which are also comprehensively analysed for their relationship with social status [4, 5, 12, 13]. As a supplementary evaluation, this paper intends to examine the relationship between social status, mental health issues and personal, social and family resources, in particular the extent to which children from socially disadvantaged families benefit from corresponding resources. Against this backdrop, we will examine three questions: (1) what is the significance of psychosocial resources for the risk of mental health issues in 11- to 17-year-old children and adolescents?; (2) are there social status-specific differences in the availability of psychosocial resources?; and, (3) how does social status affect the relationship between resources and mental health issues?

## 2. Methodology

### 2.1 Data basis

The analyses presented here build on data collected between 2014 and 2017 for the second wave of the German Health Interview and Examination Survey for Children and Adolescents (KiGGS Wave 2). The KiGGS survey has been conducted as a part of health monitoring at the Robert Koch Institute (RKI) since 2003. It also comprises repeated cross-sectional surveys of 0- to 17-year-old children and adolescents representative for Germany. Like the KiGGS baseline survey (2003–2006), KiGGS Wave 2 was conducted as a combined examination and interview survey. KiGGS Wave 1 (2009–2012) was designed and conducted as a telephone interview survey.

The population for the cross-sectional data of KiGGS Wave 2 consists of the group of 0- to 17-year-old children and adolescents with a permanent residence in Germany. Sampling was carried out via residency registration offices and the subsequent invitation of randomly selected children and adolescents from the 167 cities and municipalities of the KiGGS baseline survey. A total of 15,023 study subjects (7,538 girls, 7,485 boys) participated in the cross-sectional KiGGS Wave 2 survey. The participation rate was 40.1%. In addition, 3,567 children and adolescents participated in the screening programme (1,801 girls, 1,766 boys; participation rate: 41.5%) [14]. For the present study, 3,423 girls and 3,176 boys aged 11 to 17 years were included in the analyses.

### 2.2 Study variables

KiGGS Wave 2 recorded mental health issues based on parental responses to the Strengths and Difficulties Questionnaire (SDQ), a symptoms questionnaire comprising a total of 25 items. These refer to five subscales with five items each, namely the four problem scales Emotional Difficulties, Behavioural Issues, Hyperactivity Problems, Problems with Peers and the strength dimension Prosocial Behaviour. In this paper, however, only the four problem dimensions of the questionnaire were considered. Parents were asked to rate a total of 20 statements regarding their children. A score was calculated from the answers Not true at all (0), True to a certain extent (1) or Very true (2). Children and adolescents with a total score of up to 12 points across all areas are classified as psychologically normal, those with a score between 13 and 16 as borderline and those with a score of 16+ as presenting mental health issues [3, 12, 15]. Based on SDQ scores, respondents in the borderline and mental health issues groups were grouped together as being at risk of mental health issues [12].

Psychosocial resources were surveyed using various items and can be divided into personal, family and social resources [13, 16]. The corresponding data and results are based exclusively on self-reported data from the 11- to 17-year-old children and adolescents.

Personal resources were assessed based on a five-item scale and four possible responses for each item. These items are based on Schwarzer and Jerusalem’s self-efficacy scale (e.g. ‘for every problem I can find a solution’) [17], the Bern Questionnaire on Well-Being’ optimism scale (e.g. ‘my future looks bright’) [18] and the Children’s Sense of Coherence Scale (e.g. ‘my daily activities give me pleasure and are fun’) [19]. These questions measure personality traits such as a respondent’s sense of coherence (the feeling of being understandable, manageable and meaningful) or dispositional optimism (general confidence that things will develop positively, regardless of previous experiences). Another characteristic taken into account is general self-efficacy, i.e. the general conviction that one has the necessary skills to deal with challenges [20].

A modified version of the family health climate scale according to Schneewind et al. [21] was applied to assess family resources. This was summarised into nine items and four answers for each item. Of particular importance here are aspects of family climate, such as family cohesion and the parenting behaviour of parents (e.g. ‘we all really get along well with each other’ or ‘in our family everyone responds to the worries and needs of the others’) [20].

Social resources were assessed using a German translation of the Social Support Scale [22] with eight items. The five-stage response categories were coded with values from 1 to 5. The items ask about the social support respondents experience or that is available to them from peers and adults in the form of listening and affection, about support and help to solve problems in life as well as opportunities to do things together [20].

Overall, the item values were coded in such a way that a higher value reflects a greater resource availability. The figures were added up and transformed into values between 0 and 100. Based on an assessment of the item contents, cut-off values were determined that take into account the response distributions established in the KiGGS sample. The scale values were then divided into the categories of ‘inconspicuous or normal’, ‘below average or borderline’ and ‘significant deficits’ [13, 20]. Dummies were created for the binary logistic regressions (see 2.3 Statistical analyses). The categories ‘inconspicuous or normal’ and ‘below average or borderline’ were combined and labelled ‘medium/high’. ‘Significant deficits’ were labelled as ‘low’.

KiGGS Wave 2 records socioeconomic status (SES) based on a multidimensional index by calculating a point total score from the information provided by parents on education (school achievement and professional qualifications) and occupational status, as well as on needs-weighted net household income (net equivalent income) [23].

For each individual dimension, point values ranging from one to seven are assigned according to a fixed scheme. Information on education and occupational status is collected from the mother and father and the higher point values taken into account. In the case of single parents, the single value is used. Based on distribution, three groups are distinguished, with 20% of children and adolescents in the low-status group (1st quintile), 60% in the medium status group (2nd to 4th quintile) and 20% in the high-status group (5th quintile) [23].

A detailed description of KiGGS Wave 2 can be found in the S3/2017 issue of the Journal of Health Monitoring [16]. A more detailed description of SES is found in issue 1/2018 [23].

### 2.3 Statistical analyses

To analyse the questions described at the beginning of this article, a four-step procedure was adopted. In a first step, the distribution of mental health issues with consideration of social status was examined for 11- to 17-year-old children and adolescents. Subsequently, the distribution of psychosocial resources was examined, also segregated by social status. Psychosocial resources were always differentiated as personal, family and social resources. The third step consisted in assessing the significance of psychosocial resources for the occurrence of mental health issues. In the final fourth step, whether and, if yes, the extent to which social status affects the relationship between resources and mental health issues was examined. The analyses were carried out with the statistics programme STATA 14.2. Prevalences are presented with 95% confidence intervals. In addition, binary logistic regressions were calculated and odds ratios with 95% confidence intervals are reported. The odds ratios express the factor by which the statistical chance that the respective outcome is present is increased in a determined group in relation to a defined reference group. All calculations were carried out with a weighting factor that corrects for deviations of the sample from the general population structure with regard to age in years, gender, federal state, German nationality and parental distribution of education [24].

## 3. Results

**Figure 1:**
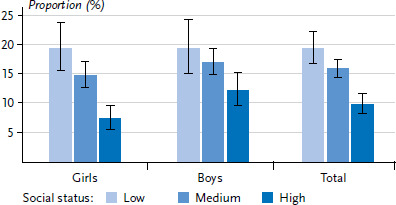
Mental health issues among 11- to 17-year-old girls and boys by socioeconomic status Source: KiGGS Wave 2 (2014–2017)

Based on the KiGGS Wave 2 data, 15.6% of 11- to 17-year-olds in Germany present mental health issues. Thereby, clear differences can be observed with regard to social status. Overall, 19.4% of 11 to 17-year-olds from the low status group present mental health issues compared to 15.9% from the medium and 9.9% from the high-status group. The social gradient is clearly evident for all genders, but is somewhat more pronounced in girls than in boys (Figure 1).

**Figure 2:**
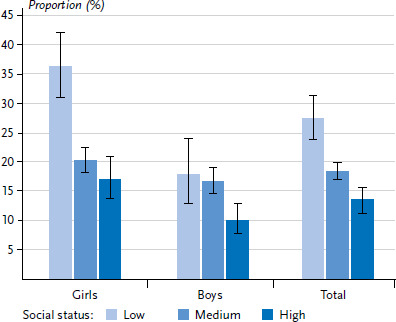
Lack of personal resources for 11- to 17-year-old girls and boys by socioeconomic status Source: KiGGS Wave 2 (2014–2017)

**Figure 3:**
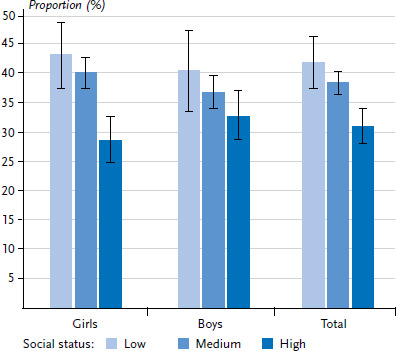
Lack of family resources among 11- to 17-year-old girls and boys by socioeconomic status Source: KiGGS Wave 2 (2014–2017)

**Figure 4:**
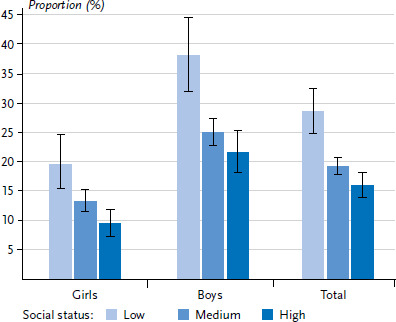
Lack of social resources among 11- to 17-year-old girls and boys by socioeconomic status Source: KiGGS Wave 2 (2014–2017)

Figure 2, Figure 3 and Figure 4 show the distribution of psychosocial resources among 11- to 17-year-old girls and boys in the different social status groups.

The results indicate that children and adolescents from the low-status group (27.3%) have few personal resources more frequently than their peers from the medium and high-status groups (18.4% and 13.3% respectively). When segregated by gender, the high proportion of girls from the low status group who have few personal resources (36.3%) is particularly striking. In the medium and high-status groups, this proportion is only about half as high. For boys, the differences are less pronounced, but still clear, at least when comparing the low to the high-status group (Figure 2).

Slightly smaller differences are observed for family resources. 42.0% of children and adolescents from the low status group have few family resources compared to 38.5% from the medium and 31.0% from the high-status group. When segregated by gender, the analyses show a somewhat more pronounced social gradient for girls than for boys. In addition, regarding the share of those with few family resources, the differences by gender are minimal and this applies to all status groups (Figure 3).

The gradient for the distribution of social resources among 11- to 17-year-olds is somewhat more pronounced (28.5% in the low-status group compared to 19.2% in the medium and 15.9% in the high-status group). This social gradient is evident for both girls and boys. Unlike for personal resources, boys score lower in social resources than girls and more often have less resources (Figure 4).

To examine the influence of psychosocial resources on mental health issues, we will first look at mental health issues in relation to the availability of resources among 11-to 17-year-old girls and boys. KiGGS Wave 2 data indicate that children and adolescents show lower levels of mental health issues overall if they have more resources at their disposal. This effect is most pronounced regarding personal resources. Here, 31.7% of the children and adolescents who have few resources evidence issues with mental health, but only 11.7% of their peers with medium/many resources. The corresponding differences in social and family resources are somewhat smaller. Of those with few social resources, 26.8% present mental health issues; of those with medium/many resources the figure is 12.6%. 21.8% of children and adolescents with few family resources have mental health issues, compared to 11.6% of those with medium/many family resources.

An analysis by gender shows that the connection between the availability of resources and mental health issues is evident as much for girls as also for boys. For personal resources, the connection is somewhat stronger for boys than for girls. For social resources, the figure for girls are somewhat greater than for boys. For family resources, the relationship is similar for girls and boys (Table 1).

Table 2 shows the relationship between psychosocial resources and mental health issues in 11- to 17-year-olds by social status. For all three resources, children and adolescents with medium/many resources are significantly less likely to present mental health issues than those with few resources. This can be observed across all three social status groups. When controlling for age and gender, children and adolescents in the low-social status group with low levels of personal resources have a 4.2-fold increased risk of presenting mental health issues compared to those with medium/many resources.

For family and social resources, too, children and adolescents with few resources more often present mental health issues than those with medium/many resources. The differences between the status groups in this regard are somewhat less pronounced than for personal resources.

When segregated by gender, a clear connection between resources and mental health issues is found for girls and boys across all status groups. Some specific aspects however do stand out. For girls, the connection between social resources and mental health issues is strongest in the high-status group. Among boys, the connection between personal resources and mental health issues is even more pronounced in the medium status group than in the low or high status group.

## 4. Discussion

For 11- to 17-year-old girls and boys, the KiGGS Wave 2 results indicate that the availability of psychosocial resources reduces the risk of mental health issues. This protective effect was visible in the analyses of personal, family and also social resources and for children and adolescents from all social status groups. At the same time, the results highlight that children and adolescents from families with low social status have fewer resources at their disposal than their peers from higher status groups and more frequently suffer mental health issues. Furthermore, a number of gender-related differences are apparent. For girls, the tie between social resources and mental health issues is somewhat stronger than for boys. On the other hand, the connection between personal resources and mental health issues is somewhat more pronounced in boys than it is in girls. However, the key finding that the psychosocial resources of children and adolescents of all status groups are associated to a reduced risk for mental health issues, applies to both girls and boys.

The results presented here are largely in line with previous research. This applies, on the one hand, to the finding of a protective effect of resources on mental health and, on the other hand, to the status-specific differences with regard to available resources and the risk of suffering mental health issues [25]. Particular reference should be made to the results of the mental health module of the KiGGS survey [26], the BELLA study (BEfragung zum seeLischen WohLbefinden und VerhAlten), which shows that children and adolescents from families with low social status more often face mental health issues and have fewer psychosocial resources at their disposal. In addition, the BELLA study showed that making use of resources reduces the risk of suffering mental health issues. Whether this applies equally to children and adolescents from all social status groups, however, has, to our knowledge, not been demonstrated in detail, neither by the BELLA study nor by other German studies [26].

In addition, international literature contains numerous studies on the links between negative experiences in childhood and adolescence, such as growing up in unstable family relationships, and impacts on health later in life. Hughes et al. [27] published a systematic review on this question, whereby 11,621 references were compiled to examine the effects of negative childhood experiences on adult health. A total of 37 studies were identified that described risk factors for 23 outcomes, such as obesity, smoking, substance abuse or mental illness. Negative childhood experiences can be a risk factor for various health outcomes later in life. Against this backdrop, the authors emphasise the importance of resilience-building and preventing negative experiences.

In their review study, Egle et al. [28] evaluate the international body of studies on the perpetuation of childhood stress experiences as well as the neurobiological and developmental psychological mechanisms that mediate these long-term consequences. They emphatically advocate for family-related prevention measures that protect parents, children and adolescents from stress and enable experiences of self-efficacy.

A number of American studies from the 1970s and 1990s are also worth referencing. In the Rochester Longitudinal Study, Sameroff et al. [29] accompanied psychologically stressed women and their children as well as an unstressed control group up to 12th grade. The Adverse Childhood Experience (ACE) study [30] was conducted by the Centres for Disease Control and Prevention towards the end of the 1990s. In two survey waves, children were examined with regard to health risks later in life as a result of negative psychological experiences in childhood. The results yielded clear evidence for a strong connection between such experiences and lifelong health consequences with effects on well-being. Compared to individuals who did not suffer adverse childhood experiences, those who suffered multiple childhood adversities (four or more ACEs) had a twice as high risk of coronary heart disease, an 1.9 times higher risk of any type of cancer, a 2.4 times higher risk of stroke, a 3.9 times higher risk of chronic lung disease and an 1.6 times higher risk of diabetes [30].

In 2019, the results of the ‘AWO-ISS Study on the long-term life course consequences of poverty’ were presented. The study focussed on the material, personal, family and social resources of children growing up in poverty in Germany. There were three survey waves with a total of 20 years of follow-up. For Germany, too, the study proves a high correlation between low social status and a limited availability of resources in childhood and adolescence with depression symptoms, low life satisfaction and need for support with drug and alcohol abuse among the now 25-year-old young adults [31]. Settings-based preventive approaches that address the overall conditions in which children grow up are listed as protective factors, for example through settings-based approaches in day-care centres and schools that aim to reduce stressors (such as bullying or situations that produce stress and pressure), strengthening resources and promoting healthier relationships between people within a respective setting. Overarching strategies to combat the consequences of poverty are identified as measures that promote health, especially in the transition between institutions and stages of socialisation (transitions), for example through municipal prevention chains [31, 32].

Various limitations must be pointed out regarding the underlying data basis and the analyses carried out. The KiGGS study uses the Strengths and Difficulties Questionnaire SDQ) to record mental health issues. However, SDQ is only a screening procedure and not a psycho-diagnostic instrument. The set age range of 11 to 17 years is large and does not take into account age group specific psychosocial health differences and the importance of personal, family and social resources. It should also be noted that the analyses were conducted based on the cross-sectional data from KiGGS Wave 2. Cross-sectionally collected data only allow statements on the relationships between the variables examined, however, not on causal relationships. Thus, for example, the question of whether the availability of resources actually reduces the risk of mental health issues, as assumed in the paper, or conversely, whether it is mental health issues that impact a person’s resources, cannot be answered conclusively. In a next step, the longitudinal data from KiGGS, which are now available, could possibly be used to answer this question [33]. It should also be pointed out that the KiGGS study uses a multidimensional index to record social status. Although this index includes data on parental levels of education and occupational status as well as on household income, other important aspects of the living situation of adolescents and their families, such as parent employment status or household composition, are not taken into account. Finally, quantitative surveys have fundamental limitations in terms of the depth of their explanations, because – unlike qualitative studies – they do not allow for a deeper understanding of individual constellations of status-related stress factors, existing resources and mental health issues.

Despite the limitations mentioned, the results point to the importance of strengthening resources as a fundamental aspect of prevention and health promotion. The results in this paper show that all children and adolescents can benefit from psychosocial resources. If resources are available, then they have a protective effect regardless of social status. However, the availability of resources is not distributed evenly across all social status groups. For this reason, measures should be identified that contribute to both reducing stress and strengthening resources in children and adolescents of all social status groups. Nonetheless, assurances would have to be made that those from socially disadvantaged families are also reached, as they will still have fewer resources. The focus should be on preventive interventions to reduce socially unequal health opportunities, for example by combating poverty, improving educational opportunities and ensuring needs-based, low-threshold counselling and support services for families under stress. In the sense of the ‘Health in All Policies’ approach, the framework conditions for children, adolescents and families could therefore be more strongly orientated towards promoting health [34, 35].

As children grow older, the importance of institutions of tertiary socialisation such as recreational child and youth facilities, sports clubs and street or school social work grows. Particularly for socially stressed young people, they offer many opportunities to strengthen resources, for example through participation, conflict resolution or other methods to promote self-efficacy. However, there are often only limited human and financial resources available for tertiary socialisation programmes. In many cases, the programmes have little conceptual, structural and financial support; accordingly, they often find it hard to retain young people [36]. In addition, in the context of the COVID-19 pandemic, maintaining such services became increasingly difficult [37]. Overall, there has been a clear increase in mental health issues, especially among young people [38]. In particular in times of crisis, however, youth outreach structures should be secured and further developed.

Preventive measures are also of great importance for example during transitions between institutions such as switching from one school to another or when people leave school (transitions), as they can counteract a spiral of resource losses and use these stations along the life course to build up psychosocial resources [36].

Overall, the relevance of personal, family and social resources described here indicates that youth outreach is an important setting for health promotion and prevention, which should be used and expanded especially to reduce socially conditioned and gender-related inequalities in health opportunities.

## Key statements

- Psychosocial resources can positively influence mental health.
- According to KiGGS Wave 2 around 16% of 11- to 17-year olds in Germany are affected by mental health issues.
- Access to psychosocial resources in society is clearly skewed, i.e. girls from the low social status group have fewer personal resources.
- The results highlight the protective function of personal, family and social resources, which calls attention to fields of action for health promotion and prevention.

## Figures and Tables

**Table 1:** Mental health issues in 11- to 17-year-old girls and boys by resources (Odds Ratios calculated using binary logistic regressions) Source: KiGGS Wave 2 (2014–2017)

	Girls				Boys				Total			
	%	(95% CI)	OR	(95% CI)	%	(95% CI)	OR	(95% CI)	%	(95% CI)	OR	(95% CI)
**Personal Resources**												
Little	26.7	(22.4–31.6)	3.2	(2.4–4.3)	38.7	(32.8–45.0)	4.2	(3.1–5.7)	31.7	(28.2–35.4)	3.7	(3.0–4.6)
Medium/Many	10.6	(8.9–12.7)		Ref.	12.7	(11.1–14.6)		Ref.	11.7	(10.6–13.0)		Ref.
**Family Resources**												
Little	20.3	(17.4–23.6)	2.2	(1.6–2.9)	23.2	(19.7–27.2)	2.1	(1.6–2.8)	21.8	(19.6–24.1)	2.4	(2.0–3.0)
Medium/Many	10.6	(8.6–13.0)		Ref.	12.5	(10.8–14.4)		Ref.	11.6	(10.2–13.1)		Ref.
**Social Resources**												
Little	28.5	(23.2–34.5)	2.9	(2.1–4.0)	26.0	(21.1–31.6)	2.3	(1.7–3.0)	26.8	(23.4–30.6)	2.6	(2.1–3.2)
Medium/Many	12.2	(10.5–14.2)		Ref.	13.4	(11.8–15.2)		Ref.	12.8	(11.6–14.1)		Ref.

OR = Odds Ratio, CI = Confidence Interval, Ref. = Reference

**Table 2:** Effects of personal, family and social resources on mental health issues in 11- to 17-year-olds by social status (Odds Ratios adjusted for age and gender) Source: KiGGS Wave 2 (2014–2017)

	Social status: Low				Social status: Medium				Social status: High			
	%	(95% CI)	OR	(95% CI)	%	(95% CI)	OR	(95% CI)	%	(95% CI)	OR	(95% CI)
**Personal Resources**												
Little	37.0	(29.4–45.5)	4.19	(2.6–6.8)	31.7	(27.1–36.6)	3.46	(2.7–4.5)	18.2	(13.1–24.7)	2.7	(1.7–4.3)
Medium/Many	13.8	(10.5–17.8)		Ref.	12.0	(10.7–13.6)		Ref.	8.6	(6.8–10.9)		Ref.
**Family Resources**												
Little	27.2	(22.1–33.3)	2.41	(1.5–3.8)	22.1	(19.3–25.1)	2.51	(2.0–3.2)	11.8	(8.5–16.1)	1.8	(1.1–2.8)
Medium/Many	14.4	(10.9–18.8)		Ref.	11.7	(10.0–13.7)		Ref.	8.1	(6.3–10.4)		Ref.
**Social Resources**												
Little	34.1	(26.6–42.4)	3.17	(2.0–5.0)	25.1	(21.1–29.6)	2.20	(1.7–2.9)	18.8	(13.6–25.4)	2.5	(1.5–4.0)
Medium/Many	14.6	(11.6–18.3)		Ref.	13.5	(11.9–15.3)		Ref.	8.1	(6.4–10.3)		Ref.

OR = Odds Ratio, CI = Confidence Interval, Ref. = Reference
